# Use of a Nutrition Behavior Change Counseling Tool: Lessons from a Rapid Qualitative Assessment in Eastern Zambia

**DOI:** 10.3389/fpubh.2016.00179

**Published:** 2016-08-31

**Authors:** Ingrid Weiss, Serena Stepanovic, Ulembe Chinyemba, Jessica Bateman, Carolyn Hemminger, Emily Burrows

**Affiliations:** ^1^University Research Co., LLC, Bethesda, MD, USA; ^2^Catholic Relief Services, Lusaka, Zambia; ^3^Catholic Relief Services, Baltimore, MD, USA

**Keywords:** counseling tool, Zambia, community nutrition promotion, nutrition agriculture integration, social behavior change

## Abstract

The U.S. Agency for International Development Feed the Future Mawa Project – led by Catholic Relief Services – aims to improve food and economic security for farming households in Zambia’s Eastern Province. Mawa employs social and behavior change (SBC) strategies with households and communities to improve nutrition and reduce stunting among children under two (CU2). To support these strategies, sub-partner University Research Co., LLC employed a participatory process to develop a series of 35 action cards, each illustrating one project-promoted behavior, that are used at household and community group levels. Caregivers of CU2 are given a full set of action cards to promote household dialogue and support for the promoted behaviors. As a final step in the action card tool development process, a qualitative rapid assessment was conducted 1 month after implementation to investigate preliminary ways action cards were being used, and if the methods of using the cards had the potential to impact behavior change. The research team conducted nine key informant interviews and four focus group discussions with Mawa staff and administered 41 qualitative interview questionnaires with project participants in the Chipata and Lundazi districts. Although not based on a representative sampling frame, the assessment produced valuable results for program improvement purposes. It also provided a feedback mechanism for community-based staff and project participants, a crucial step in the participatory tool development process. The assessment found that Mawa staff at every level use action cards combined with at least one other social behavior change tool for each nutrition intervention. Our results suggest that Mawa staff and project participants share a common understanding of the cards’ purpose. Each group noted that the cards provide a visual cue for action and reinforce previous Mawa nutrition messages. Intended uses confirmed by the assessment include encouraging household cooperation, negotiating behavior change, telling stories, and integrating messages with other project sectors. Based on the findings, recommendations for future project activities include aligning efforts against a theory of change to optimize use of all SBC tools; leveraging action card use to strengthen cross-sectoral integration within Mawa; and specific ongoing monitoring of action card use to improve activity implementation.

## Introduction

The Feed the Future (FTF) Zambia Mawa Project is funded by the U.S. Agency for International Development (USAID) and led by Catholic Relief Services (CRS) in partnership with Caritas Chipata, Women for Change, Golden Valley Agriculture Research Trust, and University Research Co., LLC (URC). The Mawa Project aims to improve food and economic security for 21,500 households in the Chiptata and Lundazi districts in Zambia’s Eastern Province. Chipata and Lundazi are two of the five districts in the FTF target region in the Eastern Province chosen by USAID based on the large number of smallholder farmers and high levels of poverty and malnutrition (1). The Mawa consortium works toward the project’s goal by providing a package of services to increase and diversify agricultural production for nutrition and markets; improve household health and nutritional status; increase incomes and productive assets; and address gender norms, roles, and beliefs related to agricultural production, household health and nutrition, and productive assets.

The project uses community-based health and nutrition promotion and support as part of its strategy to reduce stunting among children under age two (CU2) in these two districts. Stunting – a failure to reach expected height for age as a result of deficient nutritional and/or health conditions – remains far too common in developing countries like Zambia (2). Stunting is most likely to occur in the first 2 years of a child’s life and can irreversibly impact future physical and cognitive growth (3). Stunting in Zambia’s Eastern Region is slightly higher than the national average. According to the latest demographic and health survey, 43% of children under age five in this region are stunted compared to 40% nationally (4). To address the persistently high rates of stunting, evidence shows that effective programs should include a strong social and behavior change (SBC) component. Simply improving a family’s level of food security by making certain foods available does not have the desired effect of improving a child’s nutritional status unless it is accompanied by communication efforts targeting better nutrition practices (5).

The Mawa project aims to improve food and nutrition security among project participant households through an iterative process of behavior change negotiation. In order to do this effectively, the project employs the Stages of Change Model – a construct of the Transtheoretical Model. Focused on an individual’s level of intention to act, this model demonstrates that individual behavior change does not happen quickly; rather, it is a continuous process (6). One way the project supports this process is through the use of interpersonal counseling – a well-established SBC method to support the adoption of behaviors, including infant and young child feeding (IYCF) practices (7, 8). Individual and group counseling has been shown to increase exclusive breastfeeding and has demonstrated positive effects on complementary feeding practices (9, 10). Mawa implements specific SBC nutrition activities to a subset of the target population, designed to reach approximately 12,500 households over 5 years of implementation. Mawa nutrition participants are pregnant woman, caregivers to CU2, and their family members.

The Mawa project utilizes the evidence-based care group model to implement interpersonal counseling (11). The care group model is a community-based delivery strategy developed by the international non-governmental organization World Relief (12, 13). Mawa’s “care groups” are made up of nutrition volunteers, men or women who have been selected by their community to share nutrition lessons through home visits with 10 households who have a child under two or a pregnant woman. Ten nutrition volunteers making up each care group receive monthly lessons and supportive supervision from one health promoter (the group facilitator). Both health promoters and nutrition volunteers are considered to be Mawa project staff and are selected in part based upon their residence within camps where Mawa implements its integrated programing. Health promoters and nutrition volunteers contributed considerable local knowledge to the design of action cards and as part of the rapid assessment.

Mawa nutrition volunteers use several SBC job aides in their care group work. Job aides are visual images with messages that give guidance to the facilitator and have been shown to improve the understanding of the target audience (14, 15). Before the introduction of these action cards, the two primary counseling tools used by the care groups were the IYCF counseling cards and the child health reminder card. First, the nutrition volunteers use the nationally promoted IYCF community counseling cards – developed with the support of UNICEF – as a job aide to teach caregivers and communities about positive IYCF feeding and care practices (16). Second, Mawa-supported caregivers for CU2 are also given the child health reminder card – developed by the USAID Communication Support for Health project (17) – which serves as a reference for all project participant households to check how feeding practices change as their child grows. Both the IYCF cards and the child health reminder cards were developed at the national level.

### Mawa Project Action Cards

**Figure 1:**
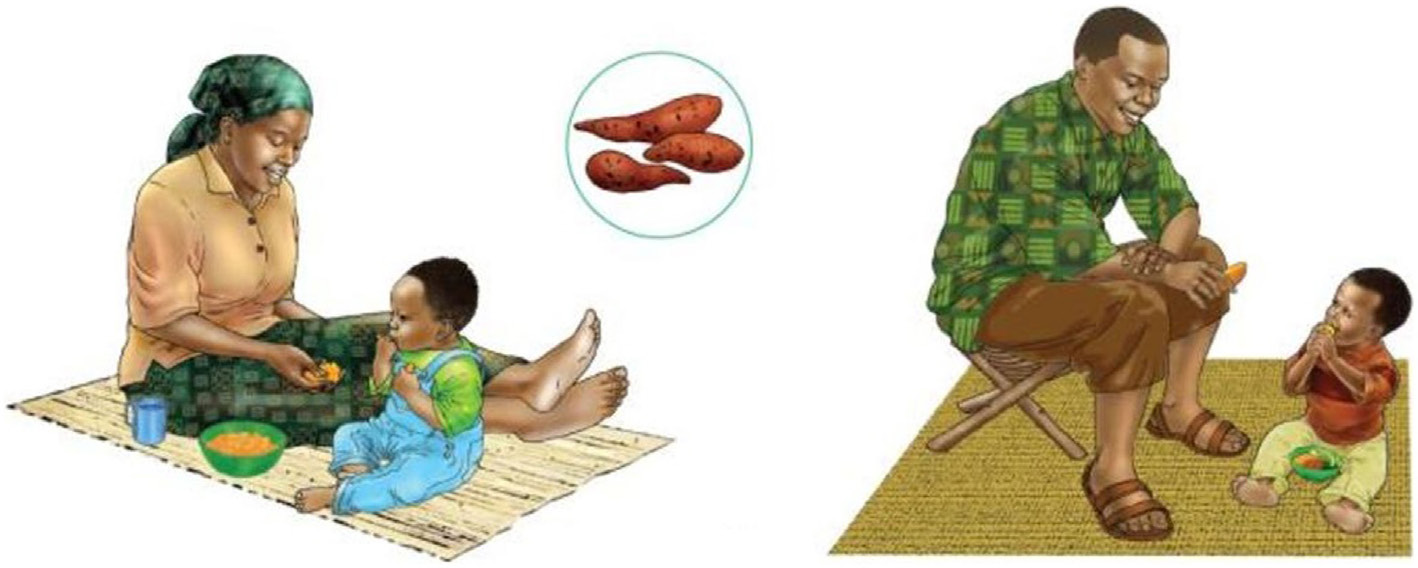
**Example action cards**.

To supplement these materials, sub-partner URC developed a series of action cards with images and actions tailored to Zambia’s Eastern Region and Mawa project participants. A set includes 35 individual action cards strung together on a binder ring. Approximately the size of an index card, each action card illustrates one simple, doable action that the project promotes to improve the health of pregnant women and CU2. Several of the cards promote behaviors that are unusual or non-traditional but still are appropriate for Mawa communities. The cards do not include any text but are numbered to allow caregivers and staff to easily reference and find cards. An example of a promoted action is mashing orange-colored foods, such as sweet potato, into a child’s porridge to improve the nutrient-density of the complementary food (see Figure 1). A set of action cards was given to each of the project-supported households with CU2 and/or pregnant women. The action cards differ from the other project SBC job aids the following ways: they do not contain text; each illustrate one action, and the actions are tailored to the Mawa project; promote nutrition-sensitive behaviors; and they are given to all caregivers.

During the initial action card training, health promoters and nutrition volunteers were asked how they would like to use the cards in lessons and in household visits; they were then presented with several different ways to use the cards. For example, staff were shown how to line up five to seven cards to tell a story, how to conduct a choice exercise by showing several cards with related behaviors and asking participants which behavior they thought they could try, and how to use the action cards to play memory or the concentration game (the concentration game is when cards are laid on their backs and one flips them over and tries to make matches). The feedback from the training was incorporated into the initial guidance on how to use the cards in the care group lessons. However, because action cards were anticipated to be used within a variety of intervention contexts, Mawa’s project leadership team also encouraged health promoters and nutrition volunteers to select and use each action card in a manner that responded to the needs of the audience – whether during household nutrition counseling sessions, community cooking demonstrations, care group sessions, or other context. This approach aimed to instill a sense of ownership over both intervention processes and outcomes among project staff, as well as to provide a mechanism for staff to actively help to shape long-term guidance on action card best practices.

Health promoters’ and nutrition volunteers’ use of the cards naturally vary by the responsibilities required of the different positions. Health promoters were encouraged to use the action cards in care group lessons to introduce new behaviors, promote key themes in lessons, and reinforce messages from the IYCF cards and the child health card. In the lessons, there are suggested action cards that correspond to the topic of the month. Often the guidance in the lessons suggests that the cards be used as a visual aid or discussion starter.

Nutrition volunteers were encouraged to use the cards to stimulate discussion of new behaviors during their household visits and to support key messages from the IYCF cards and child health reminder card, specifically by using the cards to identify small doable actions that relate to the key messages in these materials. The main purpose of the cards at this level is to help mothers select a new doable action to try for the month – a trial behavior. The nutrition volunteers negotiate with the mothers to choose a behavior and then to pick an action card that most closely matches that behavior. Next, the mothers are encouraged to discuss that card with their family during the month. A suggested best practice is to keep the card out, separate from the rest of the set, to remind mothers and their family members about the behavior they are trying that month.

At the household level, the cards are meant to encourage participation of the whole family and strengthen the voice and influence of mothers within a household. Formative research in Zambia has shown that other household influencers on IYCF, such as grandmothers or husbands, must be taken into account in nutrition interventions (18). The assumption is that the action cards can help the mother explain the selected behavior to household members, substantiate that it is promoted by the project, and facilitate household collaboration to adopt it. Owning the set of action cards may help a caregiver and/or family members feel more empowered about determining how and when to try new nutrition, agriculture, or savings practices at home (19). This notion is drawn from the Social-Ecological Model – health behaviors change when individuals’ decisions are affected by the social networks in which they function, and in order to influence lasting behavior change at the individual level, enabling environments must also be created within households, communities, and society in general (20, 21).

## Materials and Methods

The action cards were developed through a participatory process that cumulated in the rapid qualitative assessment. Starting in February 2014, Mawa engaged project participants in the conceptualization, design, pretesting, and implementation of the action cards. During the process, the intended use of the action cards was intentionally left ambiguous in order to ascertain how the participants actively adapted the cards’ use to their daily lives. The rapid assessment was the final step in the tool development process and a mechanism used to solicit feedback from project participants on how they chose to use the cards. Lessons from the development process can be used to continue building participatory processes into project implementation and research.

The rapid assessment was conducted shortly after implementation began, in case it was necessary to adapt the action card implementation plan. Including the rapid assessment as part of the tool development process ensured implementation flexibility. None of the results of the assessment should be interpreted as a critique of Mawa nutrition volunteer or health promoter performance. It was not expected that the assessment would find that staff were applying the tool smoothly, as they had limited exposure to it. Rather, it was intended to uncover their first reaction to the cards as that reaction is valuable for program design. This was a process assessment to reveal promising practices to encourage and areas of improvement at the project-level and in implementation.

### Action Card Development

In order to support individual behavior change, URC employed the Social-Ecological Model to develop the action cards. The cards were designed to create an enabling environment within households. The action cards were concept tested in February 2014. Some of the individual actions that were tested came from the UNICEF IYCF cards. During this time, families shared feedback on what actions would be helpful on cards as well as specific ways they would like to use the cards. A key finding of the concept testing was that caregivers of CU2 requested copies of the cards to have at home to share with their family members to justify and explain their new actions. While the project had been considering giving the mothers the cards, the findings from the concept testing influenced the final decision. At this stage, the project decided that the action cards should mostly feature behaviors or situations that are slightly to moderately unusual in order to stimulate the most useful discussions with household members. During the concept testing, caregivers were most interested in having cards that showed behaviors that were viewed less favorably by their family members. These cards provided additional support to caregivers for the proposed behaviors, as they were considered to be a government “sanctioned” and externally validated source of information. Another key finding that influenced the action card development was that gender images were well regarded, as they showed men and women working as a team. The images were seen as useful for sparking discussions or making a request to the husband. Men who saw the images liked them and felt that men could do more in the household if women would not mock them. During the concept testing, Mawa confirmed the purpose of the action cards was to normalize new household behaviors and strengthen the voice of mothers.

The individual actions displayed on the cards were developed using both the results of the concept testing and the findings from a positive deviance inquiry (PDI). Positive deviance is based on the observation that, in every community, there are individuals whose uncommon but successful behaviors have enabled them to find better solutions to problems than their peers who face the same challenges and have access to the same resources (22). These behaviors are likely to be affordable, acceptable, and sustainable (23). Mawa staff worked with community-based nutrition field supervisors and health promoters to review the results of the PDI and develop a list of simple, doable but less common, actions for mothers on practical feeding, caring, and hygiene to ensure healthy pregnancies and healthy growth of CU2. Some of the actions found acceptable during the concept testing were also promoted on the UNICEF IYCF community counseling cards. For example, on one of the IYCF cards, there is a sequence of images promoting exclusive breastfeeding. During concept testing, one of the images in that sequence was found to be particularly helpful and was added to the action card set.

The involvement of pertinent stakeholders in material design and testing has been shown to lead to more culturally appropriate and effective communication tools (24). As such, input from community-based nutrition volunteers suggested that more images of locally available wild foods be made available for nutrition promotion. Feedback from other Mawa sectors – agriculture, finance and savings, and gender – was incorporated into the final list of actions. This was done because Mawa’s package of interventions intentionally overlaps at the household level to build the resilience of households and to support the progression of smallholder farming families out of poverty. Key messages and actions from the other sectors are repeated in all training materials. By focusing on both the direct and underlying causes of malnutrition at the household level, Mawa aims to sustainably improve the well-being of communities.

Because participants did not express a preference for photos over drawings, the list of actions was illustrated by URC’s media department using example photos. URC had been involved in developing the illustrations for the UNICEF IYCF community counseling cards, so it was decided to use drawings as it would make the action cards look similar to the counseling cards, even though many of the illustrations were original. The hope was that the similar style of drawings would make it clear that the cards were part of the project’s package of tools, meant to be used together with the IYCF cards. Before being finalized, the action cards underwent multiple rounds of pretesting with all target audiences: mothers, fathers, grandmothers, and mothers-in-law.

### Assessment Goals and Objectives

Mawa nutrition staff began using the action cards between July and September 2015. In September 2015, Mawa conducted a rapid, participatory assessment of the early implementation of the cards, led by sub-partner URC. The assessment was participatory in that it promoted meaningful involvement of project participants, community members, and staff through structured discussion. Its intention was to provide a feedback mechanism for project staff and community members and strengthen the partnership between the project leadership, community-based staff, and community members. Assessment results present opportunities for SBC tool and strategy design and are not a critique of Mawa staff.

The rapid assessment was the final step of the participatory tool development process. The objective of the assessment was to contribute to learning on interpersonal counseling implementation strategies, specifically the current and potential role of the action cards to improve nutrition and care behaviors. The assessment investigated (1) the ways the cards were being used by Mawa staff as part of a broader suite of nutrition SBC materials; (2) the use of the cards by project participants; and (3) the potential of the cards to influence behavior change. The project used information from the assessment to adjust and improve use of the cards to better meet the needs of the community and maximize behavior change. The hope is that lessons learned from this assessment can contribute to the growing body of evidence on the process of adaptive program implementation and participatory approaches to designing SBC tools and activities.

### Research Team

The research team was comprised entirely of Mawa staff to ensure that knowledge and skills were institutionalized. The research team was large enough to allow for two data collection teams to work simultaneously. Each data collection team was composed of six members – two staff from each project sector (gender, agriculture, and savings) – to build multi-sector knowledge and cooperation. The data collection teams received training on how to collect qualitative data and a refresher SBC training. The SBC training was intended to help the team better understand the goals of the assessment and the technical content of the tools. As much as possible, Mawa involved members of the data collection teams in the data analysis. The fact that the URC team leads were based in the United States and the data collection team was in Zambia limited the involvement of the data collection teams in the final steps of the analysis.

### Sampling

The research team collected data on action card use from Mawa nutrition staff and project participant households. Assessment subjects were selected from sites where a nutrition volunteer had received and been using the action cards for at least 1 month in their community activities. The project participant subjects were caregivers of CU2 and their family members. Caregivers were defined as the person who the nutrition volunteer visited for monthly counseling sessions. For the Mawa nutrition staff subjects, the research team purposely sampled across the various levels of experience (length of employment) within the Mawa project. The research team planned a sample of staff and project participants that were equally representative across both districts. As much as possible, caregivers and family members were sampled from the same households, so households in the sample were represented by both a caregiver’s point of view and a family member’s point of view.

### Data Collection

Mawa collected data for approximately 4 days in September 2015 at four agriculture camps in the project’s two intervention districts utilizing multiple qualitative methods to triangulate information and obtain a comprehensive understanding of early action card use and its outcomes. The three assessment activities conducted were key informant interviews (KII), focus group discussions (FGD), and qualitative interview questionnaires. All research activities were conducted in the local language Nyanja in Chipata district and in Tumbuka in Lundazi district. The team immediately translated all accumulated data. Written consent was obtained from all assessment respondents for the three data collection activities.

The KIIs and FGD were conducted before the questionnaires. This allowed an iterative data collection process, where lessons learned from the KII and FGD implementation were used to refine the questionnaire instrument. Additionally, the team pretested the questionnaire, and the results were used to finalize the instrument. Data collection tools were independently translated by a staff member who was not part of the research team and checked by the data collection team – many of whom were native speakers of the local languages.

First, the research team conducted nine semi-structured KII with project implementers at all levels to identify individual thoughts, perceptions, and feelings on the use of the action cards. In each district, interviews were conducted with one project implementer per implementer category (see Table 1). To gather shared narratives on the same topics, the research team also conducted four FGD with project staff. Second, to understand both the direct and indirect experience of project participants, the research team invited a convenience sample of 21 caregivers of CU2 and 20 of their family members to participate in a qualitative, semi-structured interview. The interview questionnaire used both closed and open-ended questions to collect data on basic social and demographic data as well as general perceptions of, access to, and use of action cards. Caregiver and household data were collected from one village in Chipata and one village in Lundazi.

**Table 1:** **Assessment participants by data collection activity**.

Research method	Amount	Sample
Key informant interviews	2 interviews with nutrition volunteers	2 participants
	2 interviews with health promoters	2 participants
	4 interviews with nutrition field supervisors	4 participants
	1 interview with the nutrition team lead	1 participants
Total	9 KIIs	9 participants
Focus group discussions	2 focus groups of health promoters	12 participants
	2 focus groups of nutrition volunteers	14 participants
Total	4 FGDs	26 participants
Qualitative Questionnaires	21 semi-structured interviews of caregivers of children under age 2	21 participants
	20 semi-structured interviews of family members of caregivers of children under age 2	20 participants
Total	41 questionnaires	41 participants
	Grand total	75 participants

### Data Analysis

The analysis of the collected data followed a modified grounded theory approach [meaning that the data analysis was done without making assumptions about findings before collecting or reviewing data (25)] where written notes were analyzed by themes. A group of six project staff representing each of Mawa’s sector teams carried out the initial data synthesis and review. They were based in the assessment districts and also participated in the data collection. First, the research team completed a review of the KII and FGD transcripts and the completed questionnaires to identify initial themes. Following this, a more detailed, question-by-question review of each KII and FGD transcript was conducted by the research team leads to finalize the key themes and agree on the illustrative quotes from these activities. Data from the KIIs and FGDs were analyzed separately based on the respondent category: nutrition volunteer, health promoter, nutrition field supervisor, and nutrition team leader. Analysis for the project participant data followed the same approach but involved coding based on major identified themes, which included the perceived purpose of the cards, reported use of the cards, and implications for behavior change. The research team utilized a Microsoft-based data tabulation workbook containing the questionnaire responses summarized by frequency for each question. Caregivers and family member responses were cross-compared between questions. All original questionnaire forms, along with KII and FGD consent forms, were scanned and are stored in the URC headquarters corporate database.

## Results

Results are presented by theme, and respondent categories are broken into staff members and project participants. Staff members include the nutrition team lead, field supervisors, health promoters, and nutrition volunteers. Project participants include the caregivers and their family members. When there was a clear difference expressed by one respondent category, the category is specified. There were no notable differences in perceived purpose, use, or indicators of behavior change found between Chipata and Lundazi districts.

### Characteristics of the Study Population

Data were collected from the project nutrition team lead, 4 out of 6 field supervisors, 14 of 19 health promoters, 16 of approximate 950 nutrition volunteers, and 21 project participant households out of approximately 9,000 currently participating in nutrition activities. For the field supervisors, the average length of time in the position was 8 months. For the health promoters and nutrition volunteers, the average length of time in the position was 2 years. All of the 21 caregivers interviewed were women, with an average age of 22.5 years and an average of 2.4 children per caregiver. Of the 20 family members interviewed, 10 were men, 9 of which were husbands of the caregivers. The other types of relationships represented in the family member sample were brother, daughter, mother, mother-in-law, wife, cousin, sister-in-law, and sister. The average age of family members was 25.4 years.

### Perceived Purpose and Reported Use of Action Cards

Overall, the data suggest that Mawa staff members and participants share a common understanding of the purpose of the action cards. Respondents stated the cards served as a reminder to families of the key behaviors being promoted by Mawa. “We will use them to remind us” (caregiver, Chipata). The images themselves were described as memorable. “When they see the picture, it sticks in their minds” (health promoter, Lundazi). Similarly, the cards were frequently described as teaching tools. “…I am pregnant, so I have to learn what the cards teach” (caregiver, Lundazi). When specifically asked about the purpose of the cards, respondents less frequently cited the purpose as normalizing behaviors at the household level or promoting household discussions. However, the cards were often described in this way: “my wife told me that these cards explain to us what we should be helping each other in cooking” (family member, Lundazi). Some respondents also mentioned that the cards tell stories: “…the other benefit is that the pictures tell stories. By just seeing the picture, you are reminded of a lesson…” (health promoter, Lundazi). Responses suggest that Mawa recipients valued the perceived purpose of the cards. “I can recommend [them] because they teach us on gender [and] good nutrition to prevent malnutrition among our children” (family member, Chipata).

Mawa nutrition staff primarily use the action cards as visual aids to illustrate key points in nutrition lessons during group sessions and home visits. Nutrition volunteers who facilitate group sessions most often introduce action cards when prompted by the lesson plans. “They use the cards according to the lesson. For example, if the lesson is about the health of a child, they use the cards to show how to feed a sick child” (health promoter, Chipata). Additionally, the action cards are actively used to tell stories, primarily by nutrition field supervisors and the health promoters who facilitate the care groups. “I will pick a set of cards to make a story for teaching purposes” (health promoter, Chipata). One nutrition field supervisor from Chipata gave an example of a story that might be created with the cards, “…When the baby is born, the mother, father, and grandmother have to be present when the [nutrition volunteer] is giving a lesson (card #2) because it is mostly the grandmother who tells the mother to give the child water (#5)…and the mother, because of good [nutrition volunteer] counseling, can refuse. When the child is no longer exclusively breastfed, the mother can give the child thick porridge…” Nutrition volunteers described engaging families in playing the concentration game: “…they [nutrition volunteers] also use cards for games and stories in their spare time” (health promoter, Chipata). Nutrition staff at all levels noted the use of action cards combined with at least one other nutrition SBC tool, most frequently the IYCF counseling cards. The reported use of the cards aligns with the action card design and staff training. There were no new uses of the action cards reported in this assessment.

After the 1 month of implementation, 12 caregivers reported using the cards at home, out of the 21 caregivers surveyed. However, of the 20 family members surveyed, 14 said that the caregiver had talked to them about the action cards. It is possible that the difference in the reported use by caregivers and family members may be because some caregivers did not consider discussing the cards with family members “use.” Action card use was self-defined. If respondents reported that they used the cards, they were asked how they used them. Caregivers and family members most commonly reported using the cards to discuss the action with their family or to remind oneself or other members of the household of the key actions.

### Early Indicators of Action Card Influence on Behaviors

The perceived association between action cards and behavior adoption was strong in both the intervention districts. Both Mawa staff and caregivers believe that the action cards support behavior change because they provide a visual cue for action that reinforces earlier Mawa nutrition messages. “The behaviors are easier for the household to do because they seeing the action on the cards” (health promoter, Chipata). Mawa nutrition volunteers and health promoters mostly attributed the influence of action cards on behavior change to one point in time. Aside from being a reminder, the belief that the action cards facilitated the process of behavior change over time was expressed less frequently. “After seeing the pictures, they were convinced” (health promoter, Lundazi). This notion was also strongly associated with a perceived causal role of the Mawa project in creating behavior change among CU2 caregivers and family members through the nutrition volunteers. “To know if the [nutrition volunteers] have really taught the households, I want to see the household do the actions” (health promoter, Lundazi).

Interestingly, cards that showed actions promoting cooperation between men and women or cards that showed actions for men were popular with all respondents. These cards were often used as examples by respondents. “If me and my husband manage to work together, we will be able to have more money” (family member, Chipata). This may indicate the cards’ potential to improve gender equity and promote dialogue between men and women. In response to a question about why he liked these cards, a husband said; “On card 12, I help my wife [with] cooking food when my wife is busy with something else” (family member, Chipata).

The two key barriers for behavior change identified by participants were related to a lack of external inputs and were not related to the action cards. Occasionally, nutrition volunteers, health promoters, and project participants would describe a card inaccurately. This may suggest that they may not fully understand all of the behaviors promoted on the cards and/or that the images on the cards are not sufficiently clear, both of which are barriers to behavior change. Most field supervisors felt that health promoters needed additional guidance on how best to use the cards.

## Discussion

Among the groups sampled during this qualitative assessment, the degree of Mawa nutrition team members’ familiarity with SBC concepts and approaches was found to be the greatest among supervisors and weaker among community-based staff, the health promoters, and nutrition volunteers. This was demonstrated by community-based staff’s verbalization of behaviors changing after only one conversation at one point in time. As illustrated in the Stages of Change Model, behavior change is a process, and lasting change occurs over time. It is important for the nutrition team’s frontline change agents – the community-based staff – to have a complete understanding of behavior change and their roles in facilitating behavior change among Mawa participants. For example, using the Stages of Change Model, we are assuming that participants range between contemplation and action stages and hence can engage with each of the promoted behaviors accordingly. However, for some of the most unusual behaviors (e.g., consuming particular types of locally available protein sources, such as grasshoppers and rats), we expect that participants may be at pre-contemplation and hence will need a different type of behavioral negotiation support from nutrition volunteers. To facilitate this understanding, the project could adjust the guidance on the process of using the action cards to help nutrition volunteers keep track of preferred trial behaviors among the households that they support. Thus far, mothers seem to most often commit to adopting the behavior promoted in the current month’s lesson. In the future and to cultivate greater personal agency over process and outcomes among caregivers, the nutrition volunteer could encourage the family to make a commitment to try a project-promoted behavior of their choice for that month, and then, during the next nutrition volunteer visit, discuss the results of the trial. This would allow families to self-identify behaviors that are truly doable for them and reflect their own household priorities, sequencing, and decision-making opportunities in the coming month.

While participating in this assessment may have helped nutrition staff think more critically about the stages of change, further use of participatory research could deepen that understanding. Community-based participatory research (CBPR) is a collaborative approach to research that actively engages all the stakeholders in all aspects of the research process. CBPR can be quantitative, qualitative, or mixed-method research – it is an approach to research, as opposed to a method (26, 27). A CBPR qualitative inquiry on the process of behavior change among Mawa participants could continue to improve the project’s understanding of behavior change in project communities. Involving both Mawa staff and community members in all aspects of the inquiry would build an understanding of their complementary roles in facilitating behavior change. For the Mawa project, it would be possible to use existing community mechanism, such as neighborhood meetings (to which all community members are invited), to facilitate a qualitative inquiry. Within the context of Mawa, action cards are already used as one resource for promoting behavior change during these neighborhood meetings; Mawa could incorporate CBPR into this context by having nutrition volunteers work with households to more intentionally reflect upon and track the use of action cards as part of their behavior change process. Similarly, building upon existing practices of sharing information at the community level, the project could cultivate a tradition of storytelling based on household’s experiences with using action cards to engage in behavior change. Project staff can then capture these testimonials and stories in written form as part of an overall CBPR effort.

Designing a CBPR focused on behavior change would be a large project investment, as it would likely need to take place over a long period of time since behavior change is a lengthy process, and CBPR itself is time consuming. Additionally, it would take a significant time commitment from already heavily involved staff. However, the results from the assessment demonstrate the significant value of the process and outcomes. Ideally, in similar, new projects, a CBPR qualitative inquiry focused on behavior change could be designed in yearly project assessment efforts at the start of the project.

A complete understanding of behavior change would help staff more effectively use the suite of job aides available to them. Since the majority of staff respondents said that they used communication tools together, attention should be focused on how they use those tools in the same setting. Some health promoters did not identify a difference in the tools: “tools all say the same thing” (health promoter, Chipata). Once all Mawa nutrition team members clearly understand the purpose of each SBC tool and how best to optimize combined use of these tools within specific program implementation contexts, it is likely that the quality of the delivery of their activities, including the counseling, will increase (28). When sound theory-based interventions are not implemented with full efficiency and consistency, it can impact their effectiveness (29). Therefore, this knowledge set is important for the Mawa nutrition staff to know and apply.

As mentioned in the Section “Introduction,” the action cards differ from the other materials in three notable ways: they do not contain text; they promote nutrition-sensitive behaviors; and they are given to all caregivers. Results of the assessment demonstrate that the absence of text is a strength because less literate Mawa nutrition staff members may doubt their ability to correctly use materials with written content. “There are some people who don’t know how to read, this [the action cards] helps them grasp the messages…” (health promoter, Lundazi). A Mawa staff member also said, “The writing made people confused and doubt themselves.” Results similarly suggest that the integrated nature of the cards is a strength. Respondents made the connection between the nutrition-sensitive behaviors – especially those related to gender, agriculture, and saving – and nutrition outcomes and appeared to value the cards for the integrated behaviors. “There a lot of things that you will learn and will help us change, including gender, agriculture, nutrition” (family member, Chipata). Participants also valued the fact that the cards were given directly to project participant households for their permanent use. According to the assessment results, ownership of the cards may help the family discuss promoted practices together. The results suggest that use of the Social-Ecological Model provided a valid theory of change for the action card tool. While recognizing that caregivers exist within a multilayered, multidirectional, and complex social “ecology,” Mawa’s implementation approach to the action cards intentionally focused on the microsystem, the social family, and community networks, within which these caregivers make decisions about child feeding and personal nutrition. Mawa assumed that most caregivers were not primary household decision-makers and therefore intended for the cards to serve as external supports/validations of the non-traditional behaviors that caregivers hoped to introduce to their households. Both caregivers and family members valued the cards for this purpose.

The assessment results show that Mawa project can build on the unique strengths of the card. Several promising practices that were originally found in the concept testing were validated and expanded upon in the assessment. These practices can be emphasized at scale, especially among nutrition volunteers, caregivers, and family members. They include using the cards to encourage dialogue between health promoters, nutrition volunteers, and households and promoting more interactive use. Interactive refers to a two-way exchange of information between a counselor and participant or between a caregiver and family member, and crucially provides space for participant questions and feedback. Interactive approaches have been shown to be effective in changing nutrition behaviors (30). Under Mawa, interactive use includes using cards in games and in storytelling exercises.

Nutrition volunteers and caregivers in particular should be encouraged to continue to use the action cards to tell stories. Strategic storytelling is an evidence-based method to change attitudes and behaviors (31, 32). Compelling storytelling can create emotional appeal around the promoted actions, and emotion can be a powerful tool for instilling behavior change (33). Additionally, storytelling strengthens the voice of participants, and the process can validate the experiences of participants (34). The project can also encourage health promoters to help nutrition volunteers use the cards to promote discussions at the household level. Evidence shows that including key influencers, such as husbands and mothers-in-law, improves IYCF outcomes (35); the action cards provide a concrete way to include these household members. Staff who are currently using more interactive, participatory methods can share their experience with others to encourage wide-spread interactive use. The guidance in the lessons appeared to have a strong influence on health promoters and nutrition volunteers, so including reminders on how to use the cards more interactively in the paper lessons may promote this type of use.

### Limitations

The amount of time the cards were used by project staff and participants limited the scope of this assessment. Some staff respondents may have only used the cards in one lesson or once in a session with a caregiver. The 5-year project was in its third year when the action cards were rolled out. It was decided to implement the assessment right away, as volunteers and health promoters initiated use in order to learn from their initial reactions and recommend action based on any challenges or opportunities observed in this critical introduction and first-time-use phase. It was also necessary for the rapid assessment to take place fairly quickly after implementation to ensure implementation flexibility. Another limitation of this study was potential respondent and researcher bias. Given that the research team was comprised of Mawa project staff, health promoters and nutrition volunteers may not have been comfortable providing frank responses to their supervisors.

## Conclusion

Results from this assessment confirm the value in investigating how SBC activities are carried out using job aides (36). Results suggest that the rapid assessment had value for program improvement purposes. This assessment created a feedback loop for staff and participants and allowed the project to look at community feedback after implementation compared to community feedback from concept testing, PDIs, and pretesting. This rapid assessment was a crucial step in the participatory approach that Mawa utilized in the development and use of this SBC tool.

The results suggest that the action cards add value to Mawa’s nutrition SBC strategies. This assessment focused solely on the action cards and only collected information on the other SBC tools in relationship to the action cards. Therefore, roles of the other SBC tools in affecting behavior change cannot be derived from these results. The assessment results should not be interpreted as a comparison between Mawa’s SBC tools or that they suggest the action cards have a greater potential impact than the other tools in the behavior change process.

The results from the qualitative assessment suggest that the action cards reinforce and help to clarify messages taught by Mawa nutrition staff using other SBC tools. While the results from the assessment only describe the experiences of these respondents, these findings suggest it would be valuable for Mawa project leadership to support coaching for community-based staff to develop a more nuanced understanding of household-level behavior change. Results suggest that the project has provided Mawa staff with a clear introduction to the cards through the initial training and can now provide support on negotiation and counseling. This is in-line with lessons from past nutrition programs that show training alone is not enough to improve IYCF practices (24). Through coaching activities, the project could provide Mawa staff with support in order to better understand the behavior change process and support community-based staff to more effectively use the project’s toolkit of job aides. The first step in the coaching process should be to share the results from the rapid assessment. The Stages of Change Model, a predictive model already employed by the project, provides a useful lens through which nutrition staff can understand the process of change and their role in it. Using this model, project leadership can share simple definitions of best practices for using each tool with the community-based staff and mentor staff members to operationalize the best practices.

One of the clear strengths of the action cards is the integration of the doable actions from the other project sectors: agriculture, savings and loans, and gender. Since all care group participants are given the cards, they have become a community resource and can and should be used in the other sectors’ activities. A recommended method to share what was learned from the assessment with the community would be to use community-wide events hosted by other project sectors to share the results. Meetings held by the gender sector would be an ideal opportunity. While the assessment was the final effort for early program improvement of the action cards, additional learning should be pursued on using job aides to efficiently integrate and layer project messages, particularly with regards to gender integration and women’s empowerment.

Providing job aides to all participants is a large project investment. However, these assessment results suggest that such an investment may be worthwhile for Mawa as it may positively encourage dialogue among key household influencers about all nutrition-related behaviors. Further investigation is necessary to look at the impact of interpersonal counseling job aides that are directly provided to participants on household dialogue, communication, and decision-making over the long term. This presents an ideal opportunity to develop a CBPR inquiry and to involve community members in research design to create a collaborative and democratic strategy for generating knowledge. Additionally, a partnership approach to conduct research with community members could be used to gain a deeper insight into barriers for behavior change. The open communication and ongoing dialogue with the community that was the foundation of the action card development process could be built upon to implement successful CBPR. Lessons learned from the action card development process and the strong relationship developed with the community will make it easier to intensify and continue to use a participatory process in the Mawa project. As mentioned in the discussion session, through regular program implementation, Mawa has gained an understanding of existing community systems and structures. Mawa could use existing structures to engage community members in the inquiry process, building on local strengths and practices to facilitate a successful, participatory research process. One qualitative research tool that has the potential to be successful in CBPR in the Mawa context is the guided testimonies. A testimony is a report of one person’s experience (37). Community members could work together to develop guided testimonies about behavior change topics important to them and share these testimonies at existing community meetings.

Ultimately, this assessment improved the project’s understanding of local stakeholders, such as Mawa nutrition volunteers and supervisors, and how they interpret the purpose and use of the action cards. It also allowed community members to give feedback and participate in the implementation design. While this was the final step in the action card development process, the results suggest that collecting specific data on action card use through regular monitoring could continue to contribute to a project-wide understanding of the SBC implementation process at the community level. Results also suggest including participatory methods in monitoring action cards would increase the value of the activity. There may be an opportunity to use existing community organizing systems to introduce CBPR and involve community members in an equitable partnership to share knowledge about the process of behavior change. Knowledge gained could increase the rate of behavior change and improve nutrition outcomes among the project participants.

## Author Contributions

IW was the co-PI for the assessment, supervised data collection, conducted analysis, developed the structure of the paper, and was the lead author of the manuscript. SS was the co-PIN of the assessment, supervised data collection, conducted analysis, and contributed to writing discussions and findings. UC contributed to the assessment plan and tools, assisted in data collection and analysis, and reviewed the technical content of the manuscript. JB significantly contributed to the assessment plan and tools and participated in the original development of the action cards. CH wrote technical content for the manuscript and contributed significantly in the review process. EB, as Mawa chief of party, provided leadership throughout the process. EB reviewed and approved the assessment plan and results.

## Conflict of Interest Statement

The authors declare that the research was conducted in the absence of any commercial or financial relationships that could be construed as a potential conflict of interest. The reviewer (LG) and handling Editor declared a current collaboration, and the handling Editor states that the process nevertheless met the standards of a fair and objective review.
